# MYR1-Dependent Effectors Are the Major Drivers of a Host Cell’s Early Response to *Toxoplasma*, Including Counteracting MYR1-Independent Effects

**DOI:** 10.1128/mBio.02401-17

**Published:** 2018-04-03

**Authors:** Adit Naor, Michael W. Panas, Nicole Marino, Michael J. Coffey, Christopher J. Tonkin, John C. Boothroyd

**Affiliations:** a Department of Microbiology and Immunology, Stanford University School of Medicine, Stanford, California, USA; b Division of Infection and Immunity, The Walter and Eliza Hall Institute of Medical Research, Parkville, Melbourne, Victoria, Australia; c Department of Medical Biology, The University of Melbourne, Melbourne, Australia; Albert Einstein College of Medicine

**Keywords:** Toxoplasma gondii, effector functions, host response, host-parasite relationship

## Abstract

The obligate intracellular parasite Toxoplasma gondii controls its host cell from within the parasitophorous vacuole (PV) by using a number of diverse effector proteins, a subset of which require the aspartyl protease 5 enzyme (ASP5) and/or the recently discovered MYR1 protein to cross the PV membrane. To examine the impact these effectors have in the context of the entirety of the host response to *Toxoplasma*, we used RNA-Seq to analyze the transcriptome expression profiles of human foreskin fibroblasts infected with wild-type RH (RH-WT), RHΔ*myr1*, and RHΔ*asp5* tachyzoites. Interestingly, the majority of the differentially regulated genes responding to *Toxoplasma* infection are MYR1 dependent. A subset of MYR1 responses were ASP5 independent, and MYR1 function did not require ASP5 cleavage, suggesting the export of some effectors requires only MYR1. Gene set enrichment analysis of MYR1-dependent host responses suggests an upregulation of E2F transcription factors and the cell cycle and a downregulation related to interferon signaling, among numerous others. Most surprisingly, “hidden” responses arising in RHΔ*myr1*- but not RH-WT-infected host cells indicate counterbalancing actions of MYR1-dependent and -independent activities. The host genes and gene sets revealed here to be MYR1 dependent provide new insight into the parasite’s ability to co-opt host cell functions.

## IMPORTANCE

Toxoplasma gondii is unique in its ability to successfully invade and replicate in a broad range of host species and cells within those hosts. The complex interplay of effector proteins exported by *Toxoplasma* is key to its success in co-opting the host cell to create a favorable replicative niche. Here we show that a majority of the transcriptomic effects in tachyzoite-infected cells depend on the activity of a novel translocation system involving MYR1 and that the effectors delivered by this system are part of an intricate interplay of activators and suppressors. Removal of all MYR1-dependent effectors reveals previously unknown activities that are masked or hidden by the action of these proteins.

## INTRODUCTION

Toxoplasma gondii is an obligate intracellular parasite that infects nearly 2 billion humans worldwide. Acute infection is characterized by a high burden of rapidly replicating tachyzoites capable of infecting a broad range of hosts and a variety of cells within the body. Part of this success can be attributed to the ability of tachyzoites to establish a replicative niche in a host cell via a molecular dialogue with that cell.

At the time of invasion, the parasite uses secretion of rhoptry organelles to deliver a discrete set of ROP effectors via an ill-defined process of direct injection across the parasite and host plasma membranes and into the host cell cytoplasm (1). From the cytoplasm, these effector proteins act locally to alter actin polymerization, activate or disrupt signaling pathways such as STAT3 and STAT6, interfere in the immune response such as disrupting host GTPases, and/or traffic to the nucleus, where they are capable of directly impacting host expression (2–10).

Following invasion, the tachyzoite establishes itself in a parasitophorous vacuole (PV), whose membrane (PVM) acts as a barrier to molecular transport in and out of the vacuole. The first clues on how molecules are trafficked across the PVM were found in studies of a related apicomplexan, Plasmodium falciparum, the causative agent of malaria, where a protein translocation system called *Plasmodium* translocon of exported proteins (PTEX) employs an ATPase and actively translocates effector proteins into the erythrocyte cytoplasm (11–15). This five-component system recognizes a host-targeting (HT [16]) or *Plasmodium* export element (PEXEL [17]) motif that is cleaved by the plasmepsin V (PMV) enzyme and is required for translocation of many effector proteins using the PTEX machinery (18, 19). A set of PEXEL-negative exported proteins (PNEPs) (20), however, require the PTEX system for translocation across the PVM in an as-yet-unclear manner (11, 13). Homology with the PTEX translocation system was identified in *Toxoplasma* dense granule proteins GRA17 and GRA23; however, in *Toxoplasma*-infected cells, these proteins transport small molecules (<1,800 Da) from the host cytosol into the vacuole, rather than translocating effector proteins out (21).

Recently, we identified a *Toxoplasma* protein in the PVM that is essential for translocation of the soluble dense granule effectors GRA16 and GRA24 across the PVM (22). Because the gene encoding this novel protein was found through a genetic screen for mutants defective in host c-Myc regulation, we dubbed it *MYR1*. To date, all identified effectors that translocate dependent on MYR1 also show a requirement for the *Toxoplasma* orthologue of the *Plasmodium* PMV protease, ASP5 (i.e., aspartyl protease 5). As with much of the cargo of the *Plasmodium* PTEX system, GRA16’s export is dependent on cleavage at the so-called “*Toxoplasma* export element,” or TEXEL (23, 24). This is also true for the recently described *Toxoplasma* effector TgIST, which is involved in suppressing host cell responses to interferon signaling, also appears to have an ASP5-dependent TEXEL motif, and whose translocation across the PVM is dependent on this protease (25, 26). Although TgIST’s translocation across the PVM has not been directly analyzed in Δ*myr1* mutants, the effects it mediates are fully dependent on a functional MYR1 (22), and so like GRA16 and GRA24, this effector is almost certainly translocated in a MYR1-dependent way. GRA24, on the other hand, appears to be analogous to a PNEP protein in *Plasmodium* in not being cleaved by ASP5 but nonetheless requiring a functional ASP5 for its translocation in a MYR1-dependent manner (22–24). Other potential effector proteins have been identified that localize to the host cell nucleus, such as GRA28, and it is currently unknown how many more make use of the MYR1 translocation machinery (27).

The discovery of MYR1 allows us to probe for the first time the contribution of *Toxoplasma* dense granule effectors that cross the PVM and separate them from other effects. To do this, we have performed transcriptome sequencing (RNA-Seq) on human foreskin fibroblast (HFF) cells infected with parasites lacking MYR1 and compared the results to the response of uninfected cells as well as cells infected with either wild-type parasites or parasites lacking ASP5. We chose HFFs as the host cell because they are primary cells and have been extensively used by many in the field for transcriptomic and other analyses, allowing comparisons to that literature. We find that a substantial portion of the host genes changed during infection with *Toxoplasma* tachyzoites exhibit MYR1 dependency, and this set largely but not completely overlaps the set of host genes whose altered expression is ASP5-dependent. Consistent with this result, we also show that MYR1’s function is not dependent on ASP5. Importantly, we see effects on the host cell transcriptome not previously reported in analysis of infection with wild-type or Δ*asp5* mutants, indicative of MYR1-independent activities, including, but not limited to effector proteins, pathogen-associated molecular patterns, and the effects of cellular trauma, whose impacts are normally countered by MYR1-dependent effector proteins. The results presented also indicate that there are apparently important, yet-to-be-identified effectors whose activity is dependent on MYR1 but not ASP5.

## RESULTS

### Establishing conditions and analytic methods for RNA-Seq on infected cells.

To assess the totality of the impact that MYR1-dependent effectors have on the host, we chose RNA-Seq as a powerful method for determining global changes in host cells infected with wild-type and Δ*myr1* mutant tachyzoites. Using a 6-hours-postinfection (hpi) time point allowed us to capture the transcriptional changes that result from both rhoptry and dense granule effectors, while minimizing the number of secondary transcriptional changes that might be downstream effects. The HFFs were infected with either wild-type RH (RH-WT), RHΔ*myr1*, or the complemented strain RHΔ*myr1*::*MYR1* or mock infected. Mock infection involved addition of a parasite-free cell lysate to mimic the cellular debris created when parasites are freed from their host cells, thus controlling for the impact of host cell damage-associated molecular patterns. Infection at a high multiplicity of infection (MOI) of 5 was chosen to generate the highest percentage of infected cells without producing lysis of the host cells by the 6-h harvest point. We performed three biological replicates for this experiment, each with either two or three technical replicates, and mapped the reads to the human genome (Table 1). These biological repeats were varied in the month they were performed and the stock of human foreskin fibroblasts used as host cells. Each biological experiment involved RNA extraction, library preparation, and sequencing together, but separate from the other biological repeats, allowing a better direct comparison between samples. These numerous replicates allowed us to set the statistical significance for what to call “changed” as having a stringent *q* value of 0.05 and false-discovery rate (FDR) set at 10%, in addition to a threshold of 1.5-fold change up (upregulated) or down (downregulated).

**TABLE 1:** Summary of total RNA-Seq reads mapped

Infection and sample no.	No. (%) of reads mapped^a^	
	Human genome	*Toxoplasma* genome
Mock		
1	28,303,437 (99.5)	136,537 (0.5)
2	26,656,452 (99.4)	153,263 (0.6)
3	13,637,605 (99.2)	105,372 (0.8)
4	13,007,385 (99.3)	98,286 (0.7)
5	5,280,417 (99.2)	40,551 (0.8)
6	15,675,192 (99.5)	85,248 (0.5)
7	10,225,078 (99.3)	68,095 (0.7)

RH		
1	17,541,338 (76.3)	5,436,742 (23.7)
2	15,710,664 (72)	6,111,477 (28)
3	13,401,169 (71.5)	5,352,720 (28.5)
4	13,606,269 (75.2)	4,497,829 (24.8)
5	1,895,604 (67.1)	929,937 (32.9)
6	1,501,296 (77.5)	436,416 (22.5)
7	1,118,902 (63.9)	631,314 (36.1)

RHΔ*myr1*		
1	18,334,239 (78.3)	5,080,247 (21.7)
2	17,603,181 (72.7)	6,625,567 (27.3)
3	12,889,729 (64)	7,253,952 (36)
4	15,546,151 (71.9)	6,078,441 (28.1)
5	3,996,692 (63.7)	2,276,057 (36.3)
6	1,246,788 (68.1)	583,369 (31.9)
7	2,284,809 (61.8)	1,410,089 (38.2)

RHΔ*myr1*::*MYR1*		
1	18,645,106 (79.3)	4,864,519 (20.7)
2	17,179,730 (72.2)	6,629,830 (27.8)
3	13,579,524 (67.5)	6,531,328 (32.5)
4	8,443,874 (69.5)	3,713,665 (30.5)
5	5,650,821 (79.2)	1,488,299 (20.8)
6	3,624,860 (52.3)	3,299,922 (47.7)
7	1,409,132 (49.3)	1,451,964 (50.7)

RHΔ*asp5*		
1	10,592,764 (87.4)	1,523,876 (12.6)
2	10,290,292 (75.1)	3,411,275 (24.9)
3	5,057,673 (76.8)	1,523,876 (23.12)
4	7,498,195 (77.9)	2,121,971 (22.1)
5	3,978,935 (75.5)	1,291,803 (24.5)

RHΔ*asp5*::*ASP5*		
1	10,177,793 (65.2)	5,433,843 (34.8)
2	9,791,658 (81.4)	2,235,700 (18.6)
3	3,690,117 (63.5)	2,123,616 (36.5)
4	6,088,041 (86.9)	921,158 (13.1)
5	5,679,354 (82.4)	1,216,067 (17.6)

a Shown are the numbers of raw reads mapped to human and *Toxoplasma* exons using CLC Genomics and the percentage of all reads mapping to these genomes.

To determine the impact that MYR1-dependent translocation has on the host response, we performed the RNA-Seq analysis in two different ways. In the first approach, the responses that tachyzoites engender were identified by comparing either an RH-WT or an RHΔ*myr1* infection to the mock-infected state. These two sets of differential responses were then compared to each other, and responses arising in both were designated MYR1-independent, while those arising in only the RH-WT-infected cells are suggested to be MYR1-dependent. Because this approach only compares lists of genes with altered expression, it misses quantitative changes in host gene expression that are, for example, significantly higher in both sets of infected cells (RH-WT and RHΔ*myr1*) relative to mock infected, but to markedly different degrees (e.g., 2-fold upregulated in RH-WT-infected cells and 7-fold upregulated in RHΔ*myr1*-infected cells). To overcome this limitation, we also used a second approach that directly compares gene expression levels in the RH-WT versus RHΔ*myr1*-infected cells. Note that the latter approach lacks the ability to identify MYR1-independent responses, where infection with both parasite lines produces the same change (e.g., both 3-fold upregulated) relative to mock-infected cells. Together, however, these two approaches should reveal the full spectrum and degree of MYR1-dependent and -independent effects, and so both were used here. To represent the data throughout the article, we have chosen heat maps that display the lowest expression level for a given gene across all samples as blue and the highest expression as red, with all other values as intermediate colors along a log_2_ continuum between the two extremes. As a consequence of this, for genes where the boundary values are relatively close (e.g., varying between a value of 100 and 150 reads per kilobase per million [RPKM]), relatively small differences in expression levels appear as large differences in color. This allows us, however, to graphically present in one image the data for all genes, regardless of their absolute RPKM values (which range from 0 to >13,000) and their fold differences (which range from the minimum we imposed of 1.5-fold to >150-fold for the most dramatically affected genes).

### RNA-Seq of infected cells shows MYR1 is essential for a large portion of the host cell’s transcriptomic response to infection.

**FIG 1:**
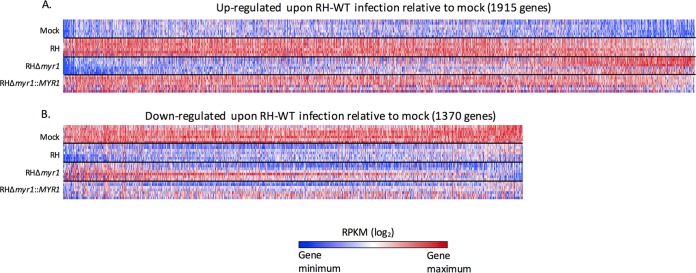
Host transcriptomic responses 6 h postinfection are largely MYR1-dependent. Host RNA expression levels in mock-infected HFFs were compared to expression levels of HFFs infected with wild-type RH, RHΔ*myr1*, and RHΔ*myr1*::*MYR1* tachyzoites. (A) Heat maps displaying expression levels for the 1,915 genes that in the RH-WT-infected cells exhibited a statistically significant increase (*q* value of <0.05, 10% FDR) of ≥1.5-fold over the expression in mock-infected HFFs. (B) As for panel A, except expression levels are for the 1,370 genes that exhibited a statistically significant decrease of ≥1.5-fold relative to mock infection. Gene names and average RPKMs can be found in Table S1. Seven replicates are shown for each of the four conditions (uninfected and three parasite-infected lines) with the rows 1 to 3, 4/5, and 6/7 being technical replicates of the first, second and third biological experiments, respectively. The log_2_ values for all 28 data points for each gene are colored red for the highest value and blue for the lowest value, regardless of the range of values they represent. All heat maps display genes sorted from left to right on the basis of decreasing magnitude of the average difference in expression between cells infected with RH-WT versus RHΔ*myr1*.

Using the first approach, we compared results from the seven replicates of RH-WT-infected cells with the seven replicates of mock-infected cells and identified host genes whose transcript abundances were significantly different under the two conditions. This set of genes defines the total transcriptomic response to tachyzoites at 6 h. Consistent with previous publications (5, 10, 28–30) and using the criteria stated above, we found that the expression of 3,285 host genes was changed, representing ~12% of the 27,401 genes that were detectably expressed (Fig. 1). Within this group of 3,285 genes, 1,915 demonstrated an increase in expression upon infection (Fig. 1A; see Table S1A in the supplemental material), with fold differences relative to mock infected varying over 2 orders of magnitude. In contrast, 1,370 genes’ expression was downregulated with, as expected, a much lower range of impact due to the presence of uninfected cells in the “infected” cultures that contribute reads (Fig. 1B; Table S1B). For example, with ~10% of cells uninfected in these cultures, even a gene whose transcripts completely disappear in infected cells will still show a value of 10% relative to mock infected and, therefore, a fold change of at most 10-fold (assuming no effect of infected cells on the uninfected cells).

10.1128/mBio.02401-17.2 TABLE S1  Host transcriptomic responses 6 h postinfection are largely MYR1-dependent. The table provides the average RPKMs for all of the host genes in the heat maps shown in Figure 1. Download TABLE S1, XLSX file, 0.3 MB. Copyright © 2018 Naor et al. 2018 Naor et al. This content is distributed under the terms of the Creative Commons Attribution 4.0 International license.

Next, and in the same manner as described above, the impact of infection with RHΔ*myr1* was determined by comparing transcript levels with those in mock-infected cells. As a control, infection with a complemented version of this mutant, RHΔ*myr1*::*MYR1*, followed by RNA-Seq was also performed (Fig. 1; Table S1A and B). Effective complementation was demonstrated by comparing the RPKM ratios for all host genes that were significantly different in RHΔ*myr1*- versus RH-WT-infected cells with the ratios for those genes in RHΔ*myr1*- versus RHΔ*myr1*::*MYR1-*infected cells. The results showed that the majority of the differences seen in the RHΔ*myr1-*infected cells were indeed rescued by complementation (see Fig. S1A in the supplemental material), confirming that the results observed upon deletion of the *MYR1* gene were due to that disruption, not an independent, spurious mutation.

10.1128/mBio.02401-17.1 FIG S1  A comparison of the RHΔ*myr1*::*MYR1* and RHΔ*asp5*::*ASP5* mutants demonstrates that many MYR1- and ASP5-dependent genes are rescued by complementation. (A) When the MYR1-dependent genes are analyzed using the same threshold of 1.5× and statistically significant cutoff applied throughout the article, 59% of these genes are returned to their wild-type values by complementation. All 1,648 host genes shown to be changed in a MYR1-dependent manner are plotted. The ratios of the host genes’ RPKM values during infection with RH versus the value during infection with RHΔ*myr1* are compared to the ratios of the RPKM values during infection with RHΔ*myr1*::*MYR1* versus infection with RHΔ*myr1*. (B) All 713 host genes changed in an ASP5-dependent manner are plotted. The ratios of the host genes’ RPKM values during infection with RH versus the RPKM values during infection with RHΔ*asp5* are compared to the ratios of the RPKM values during infection with RHΔ*asp5*::*ASP5* versus infection with RHΔ*asp5*. Download FIG S1, TIF file, 1.7 MB. Copyright © 2018 Naor et al. 2018 Naor et al. This content is distributed under the terms of the Creative Commons Attribution 4.0 International license.

**FIG 2:**
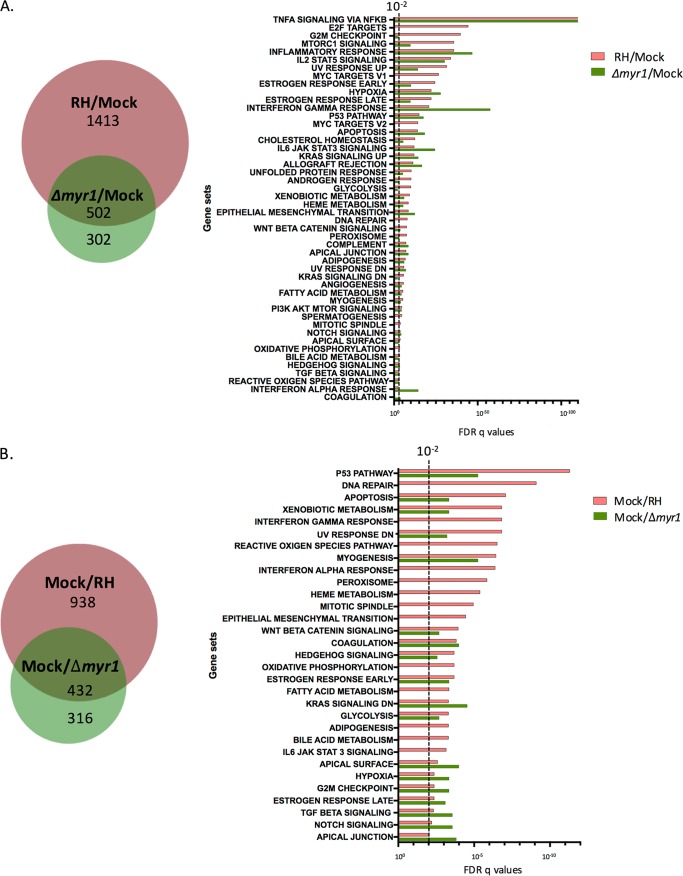
Comparison of gene sets affected in a MYR1-dependent manner. (A) The overlap of host genes significantly upregulated ≥1.5-fold during infection with RH and with RHΔ*myr1* compared to mock infected, as displayed in Fig. 1, are summarized in a Venn diagram. These genes were analyzed by gene set enrichment analysis (GSEA), and the FDRs for each significantly affected (*q* ≤ 0.05) gene set are displayed on the right. The dotted line represents an FDR *q* value of 10^−2^. Note that GSEA only provides data for gene sets with *q* values of ≤10^−2^, so values of >10^−2^ are not shown. (B) As in panel A, except showing overlap of genes downregulated during infection with RH and RHΔ*myr1* compared with mock infected. Complete lists of the genes contributing to the GSEAs shown in Fig. 2A and B are given in Tables S4 and S5, respectively.

Having validated the data for the RHΔ*myr1*-infected cells, we were able to next determine which transcriptomic changes upon infection with RH-WT result from MYR1-independent effects. We did this by comparing the list of host genes altered in RH-WT-infected cells relative to mock infection with the list of host genes altered in RHΔ*myr1-*infected cells relative to mock infection. The results revealed that only 502 (26%) of the 1,915 genes that were upregulated (based on both statistical significance and a ≥1.5× change) upon infection with RH-WT were also upregulated in cells infected with RHΔ*myr1* (Fig. 2A, left). Similarly, only 432 (32%) of the 1,370 host genes that were downregulated in RH-WT-infected cells were also downregulated in RHΔ*myr1*-infected cells (Fig. 2B, left). These overlaps represent effects that are independent of MYR1, and these could be mediated by rhoptry-derived ROP proteins or dense granule effectors such as GRA15 and MAF1 that operate at, but do not fully cross, the PVM and are therefore MYR1-independent (22). MYR1-independent factors could also include triggers such as pathogen-associated molecular patterns or cellular damage caused by the trauma of an invading body. The fact that only 26% of upregulated and 32% of downregulated responses can definitively be shown to be MYR1-independent strongly suggests that MYR1-dependent responses comprise a large portion of the total response. Interestingly, the results also revealed the existence of host genes whose expression is different in the RHΔ*myr1*-infected cells but not in the RH-WT-infected cells, both relative to uninfected cells and represented by the pure green areas of the Venn diagrams shown in Fig. 2. This class of “hidden” effects is discussed further below.

To begin to understand the impact of the MYR1-dependent and -independent transcriptomic changes and to infer possible mechanisms underlying them, we applied gene set enrichment analysis (GSEA) (31) on the 1,915 genes that are upregulated during RH-WT infection and on the 804 that are upregulated during RHΔ*myr1* infection. GSEA compares results obtained under different experimental conditions in different laboratories on a variety of cell types and using a variety of means (genetic through chemical) to produce the perturbations. It therefore yields clues to pathways that may be affected but does not give unambiguous results about the precise factors or sequence of events involved. The GSEA results (Fig. 2A; see Table S4 in the supplemental material) showed strongest enrichment in the genes upregulated in both the RH-WT and RHΔ*myr1* infections for the set of genes previously shown to be altered by tumor necrosis factor alpha (TNF-α) signaling and entirely consistent with the action of the MYR1-independent GRA15 (22, 32). Conversely, the gene sets associated with the E2F and c-Myc transcription factors, G_2_M checkpoint, and glycolysis were all significantly enriched in cells infected with RH-WT relative to mock infection but not in cells infected with RHΔ*myr1*, demonstrating a dependence for these effects on MYR1. Some of these latter effects were predicted because published KEGG analysis of the microarray data generated in analyzing the response to MYR1-dependent GRA16 showed involvement of this protein in p53 and cell cycle pathways as well as in pathways related to nutrient metabolism (33).

We also performed GSEA on the sets of downregulated genes, i.e., 1,370 genes that were downregulated between RH-WT versus mock infected and 748 genes that were downregulated between RHΔ*myr1* versus mock infected (Fig. 2B; see Table S5 in the supplemental material). The results showed a substantial impact of infection with RH-WT on gene sets associated with perturbations in checkpoint control, such as the p53 pathway, DNA damage, apoptosis, the UV response, and cytokine signaling, such as interferon responses and JAK/STAT signaling. When analyzing these gene sets, the majority were either not significantly different in the RHΔ*myr1*-infected cells relative to mock infected or were seen with a markedly higher FDR *q* value, indicating they are at least partially MYR1-dependent effects. These included gene sets related to type I and type II interferon responses, apoptosis, and interleukin-6 (IL-6)–JAK/STAT pathways, all of which would be predicted from the GSEA of host cells infected with the TgIST knockout (25), or in the case of the IL-6–JAK/STAT pathway, the KEGG analysis of the GRA24 knockout microarray data set (34). We observed that the GSEA on the downregulated genes generally identified sets with a much lower significance value than in the upregulated set, with a *q* value of 2e−11 for the most significantly downregulated gene set (p53 pathway) versus 1.9e−111 for the most significantly upregulated gene set (NF-κB) (Fig. 2A). This is likely due, in part, to the aforementioned impact of uninfected cells in the infected cultures.

**FIG 3:**
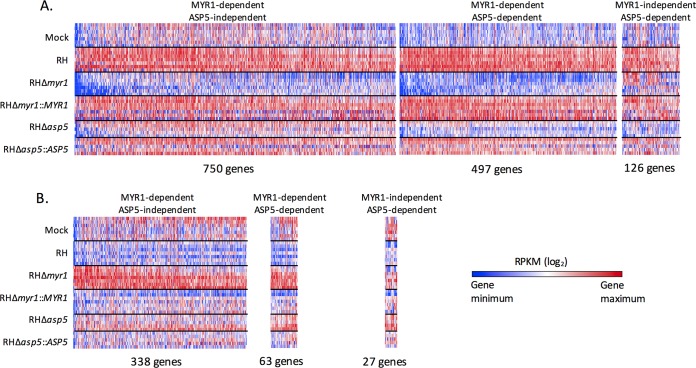

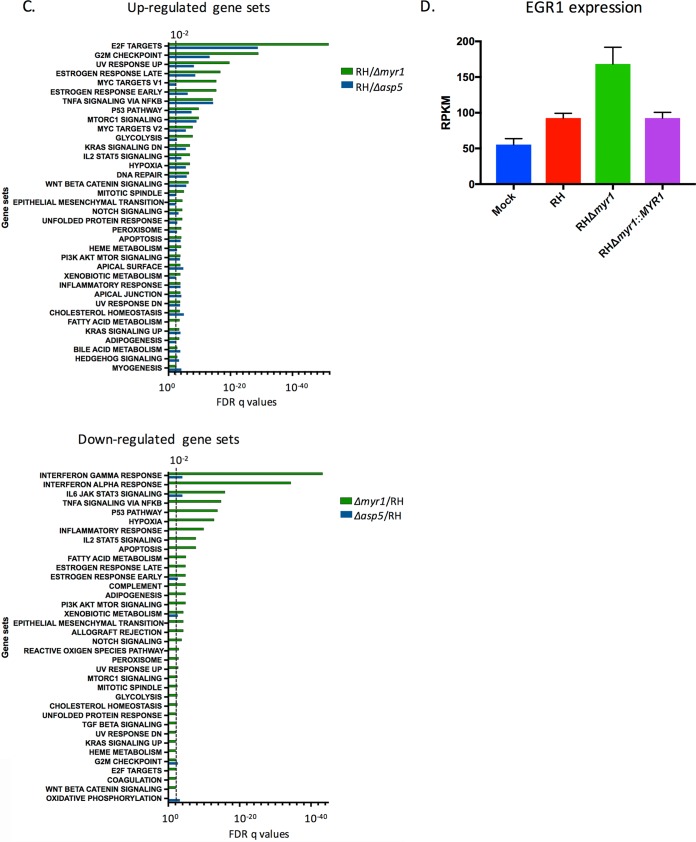
Comparison of genes differentially expressed in a MYR1- and ASP5-dependent manner compared to wild-type infection. Host gene responses are shown that are statistically significantly increased ≥1.5-fold (A) or decreased ≥1.5-fold (B) in cells infected with wild-type RH compared to RHΔ*myr1* or RHΔ*asp5*. Genes are considered MYR1- or ASP5-dependent if their change between RH and the respective knockout met the threshold of a *q* value of <0.05, 10% FDR, and ≥1.5-fold change, and were considered independent of these two parasite proteins if the differential gene expression did not meet that threshold. The first panel shows all genes that were significantly different in RHΔ*myr1*- but not RHΔ*asp5*-infected cells relative to RH-WT infection, the middle panel shows genes that were different in both mutants relative to RH-WT, and the third panel shows genes that were different in only the RHΔ*asp5*-infected cells (not RHΔ*myr1* infections) relative to RH-WT. Genes in the first two panels are arranged from left to right in decreasing magnitude of difference between RH-WT- and RHΔ*myr1*-infected cells, and genes in the third panel are arranged in decreasing magnitude of the difference between RH-WT and RHΔ*asp5* infection. (C) MYR1-dependent and ASP5-dependent host genes were analyzed by GSEA, and the gene sets are displayed with a threshold of an FDR *q* value of <10^−2^. Complete lists of the genes contributing to the GSEAs shown for up- and downregulation are given in Tables S6 and S7, respectively. All other details are as in Fig. 2. (D) RPKM expression values of the EGR1 (early growth response 1) gene of HFFs infected with the indicated parasite lines. Error bars represent standard error of the mean (SEM).

GSEA comparisons between RH-WT-infected or RHΔ*myr1-*infected cells relative to mock infected will miss some of the MYR1-dependent effects because they only look at gene lists, independent of the magnitude of the transcriptional changes. Thus, compared to the mock-infected cells, a host gene that is expressed 10-fold and 2-fold higher in the RH-WT- and RHΔ*myr1-*infected cells, respectively, shows up in both lists, thus making it MYR1-independent by the above definition, yet obscuring the fact that the dramatic change seen in the RH-WT infection is largely (though not entirely) dependent on MYR1. To address this limitation, we performed a direct comparison of the RNA-Seq data from infection with RH-WT versus RHΔ*myr1*. The results (Fig. 3A and B, which also include data for ASP5 dependency discussed below; see Table S2 in the supplemental material) showed 1,247 (750 + 497) host genes whose transcripts were significantly higher and 401 (338 + 63) whose transcripts were significantly lower in RH-WT-infected versus RHΔ*myr1*-infected cells. These represent changes that are dependent on a functional MYR1. GSEA of these MYR1-dependent effects (Fig. 3C) revealed an apparent contradiction in that one predominant GSEA gene set, “TNF-α signaling via NF-κB,” was the most strongly overrepresented in the set of genes upregulated in both infections (RHΔ*myr1* and RH-WT) compared to mock infected, suggesting MYR1 independence (Fig. 2A), and yet the same gene set was also significantly different in the head-to-head comparison, suggesting some dependence on MYR1. To resolve this, we examined the identities and individual expression levels of the actual genes in each “TNF-α signaling via NF-κB” set. The overlap in genes between these lists was small: i.e., different genes were representing the “TNF-α signaling via NF-κB” gene set under each of these three conditions. This examination also showed examples where substantial differences in the magnitude of the effect were seen: i.e., the same gene was present in two or more comparisons but with marked differences in the degree of change (Table S2A to F). An example of the latter is the EGR1 (early growth response 1) gene (Fig. 3D), whose average RPKM expression levels were 55 in mock infection, increasing to 93 (up 1.7-fold) in the cells infected with RH-WT, even higher (168; 3-fold above mock infected and 1.8-fold above RH-WT infected) in the RHΔ*myr1*-infected cells, and back down to 93 (1.7-fold) for RHΔ*myr1*::*MYR1*. Thus, the factor or factors responsible for EGR1’s upregulation upon infection appear to include both MYR1-dependent and MYR1-independent factors.

10.1128/mBio.02401-17.3 TABLE S2  Comparison of gene sets affected in a MYR1-dependent manner. (A and D) MYR1 dependent, ASP5 independent. (B and E) MYR1 dependent, ASP5 dependent. (C and F) MYR1 independent, ASP5 dependent. The table provides the average RPKMs for all of the host genes in the heat maps shown in Figure 3. Download TABLE S2, XLSX file, 0.2 MB. Copyright © 2018 Naor et al. 2018 Naor et al. This content is distributed under the terms of the Creative Commons Attribution 4.0 International license.

### MYR1-dependent effectors counter the impact of MYR1-independent factors.

**FIG 4:**
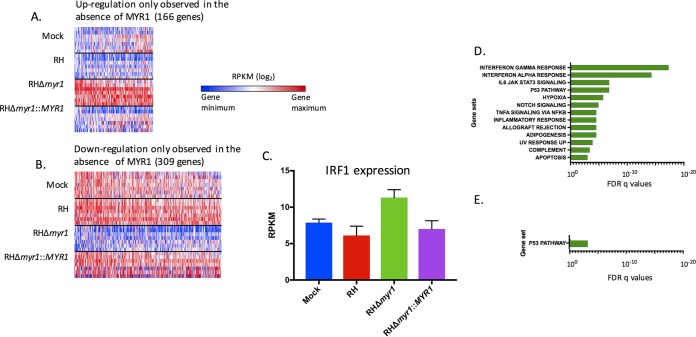
Parasite-induced gene expression changes that are masked in a MYR1-dependent manner. (A) Heat map of the 166 genes that exhibit a statistically significant higher expression of ≥1.5-fold between RHΔ*myr1* and RH-WT-infected cells, but not between mock- and RH-WT-infected cells. (B) As for panel A, except the heat map shows the 309 genes that exhibit a statistically significant lower expression of ≥1.5-fold change between RHΔ*myr1*- and RH-WT-infected cells, but not between RH-WT- and mock-infected cells. (C) Average RPKM values for the IRF1 gene in the different infections. (D) GSEA on the genes from panel A. (E) GSEA on the genes from panel B. Complete lists of the genes contributing to the GSEAs shown in Fig. 4A and B are given in Table S8. All other details for panels D and E are as in Fig. 3C.

The direct comparison between RHΔ*myr1*- and RH-WT-infected cells allowed us to also ask if there might be instances in which a MYR1-dependent effector counteracts the effect of a MYR1-independent factor such that the net effect in an RH-WT-infected cell is no change to a given host gene’s expression. The data in Fig. 4 show that, in fact, 166 host genes were specifically upregulated in the RHΔ*myr1-*infected cells relative to both RH-WT- and mock-infected cells (Fig. 4A; see Table S3A in the supplemental material) and 309 were specifically downregulated (Fig. 4B; Table S3B). Conceptually, these genes represent the same gene populations as those in the “green-only” area of the Venn diagrams of Fig. 2 that are differentially changed in the RHΔ*myr1* versus mock infection comparison but not in the RH-WT versus mock infection comparison; however, because the set of genes in Fig. 4 was derived first by analyzing RHΔ*myr1*- versus RH-WT-infected cells before then comparing those results to mock infected, the threshold and statistical cutoff generate somewhat different populations.

10.1128/mBio.02401-17.4 TABLE S3  Parasite-induced gene expression changes that are masked in a MYR1-dependent manner. The table provides the average RPKMs for all of the host genes in the heat maps shown in Figure 4. Download TABLE S3, XLSX file, 0.1 MB. Copyright © 2018 Naor et al. 2018 Naor et al. This content is distributed under the terms of the Creative Commons Attribution 4.0 International license.

One example of a host gene whose expression changes only in the RHΔ*myr1-*infected cells is the interferon regulatory factor 1 (IRF1) gene, which has been well studied by others in the context of *Toxoplasma* infection (35–37). This gene’s transcripts did not significantly differ from the mock-infected value during infection with RH-WT, but in the RHΔ*myr1*-infected cells, the transcript level was significantly different at 1.9-fold higher than in the RH-WT-infected cells, returning to wild-type levels when RHΔ*myr1* was complemented with a wild-type copy of MYR1 (Fig. 4C). This suggests that a MYR1-dependent suppressor normally counteracts the effect of a MYR1-independent agonist that would otherwise stimulate IRF1 expression. Candidate molecules for these effects are discussed further below.

To explore the full range of counteracting effects, we applied GSEA to the genes that, like the IRF1 gene, had expression levels that were not significantly different from mock infected in the RH-WT-infected cells but were upregulated in RHΔ*myr1-*infected cells. This analysis showed the greatest enrichment was in gene sets related to type I and type II interferon responses, consistent with the data for IRF1 (Fig. 4D; see Table S8A in the supplemental material). The set of genes that exhibit no significant change in RH-WT-infected cells relative to mock infected but were lower in RHΔ*myr1-*infected cells may be examples of genes whose expression is normally subject to activation via MYR1-dependent effectors and suppression by MYR1-independent factors; in the absence of MYR1: therefore, only the suppression effect is seen. Interestingly, however, GSEA of these data shows enrichment only in p53-related pathways and only to a very low significance score (10e−3.2 [Fig. 4E; Table S8B]), suggesting that there are relatively few examples of strong MYR1-dependent activators countered by MYR1-independent suppressive effects.

### MYR1-dependent effects on the host are substantially more numerous than the ASP5-dependent effects.

Previous work has shown that the *Toxoplasma* aspartyl protease 5 protein (ASP5, a Golgi apparatus-resident enzyme) cleaves a subset of secreted proteins and is required for the export of the three known nonrhoptry effector proteins that reach the host nucleus: GRA16, GRA24, and TgIST (23, 25). As ASP5 also cleaves MYR1, it was possible that the requirement for this enzyme for export was mostly or entirely due to a role for it in activation of MYR1. If that was so, then the effect of the loss of ASP5 on the host cell transcriptome, shown previously to be substantial, should overlap largely or even completely the response seen in the absence of MYR1. To test this, we added RHΔ*asp5* and its complemented control, RHΔ*asp5*::*ASP5*, to the conditions subjected to RNA-Seq analysis (Fig. 3; Tables S3A and B, respectively). As for the RHΔ*myr1* mutant, we first checked whether complementation with the wild-type ASP5 allele rescued the phenotype of the RHΔ*asp5* mutant. The results (Fig. S1B) showed that the majority of transcriptomic differences seen in the RHΔ*asp5* mutant appeared to be substantially rescued by the complementation.

Having validated the RHΔ*asp5* data, we next compared the host responses by their ASP5 dependency versus their MYR1 dependency. To do this, we performed a direct comparison of the data for these mutants with the data for RH-WT infection and sorted the results based on host genes that are significantly changed between infection with RH-WT and RHΔ*asp5* and compared this to the data for RH-WT versus RHΔ*myr1*. As shown in Fig. 3A, there were 623 host genes whose upregulation was ASP5 dependent: i.e., that were significantly higher by ≥1.5-fold in RH-WT-infected versus RHΔ*asp5*-infected cells. The overlap between these 623 genes and the MYR1-dependent sets was 497, or 80%. This overlap of 497, however, represented only 40% (497/1,247) of the total MYR1-dependent response. A similar trend was observed for the host genes that show a lower expression upon infection with the mutants relative to RH-WT (Fig. 3B), where just 90 showed ASP5 dependence, with 63 (70%) also being MYR1-dependent, and these 63 represent just 16% of the total of 401 host genes showing MYR1-dependence. These results indicate that there are likely major effector proteins that require the activity of MYR1 but not ASP5 for export. GSEA of the ASP5-dependent data showed no gene set that was dependent on ASP5 that was not also dependent on MYR1 in the gene sets that were expressed higher (upregulated) in the RH-WT-infected cells than in the two mutants and only one (oxidative phosphorylation) in the gene sets that were expressed lower (downregulated) (Fig. 3C; see Tables S6 and S7 in the supplemental material). This result again indicates that while many effects appear dependent on MYR1, only a subset of these is dependent on ASP5.

### ASP5-mediated cleavage of MYR1 is not essential for MYR1’s role in export.

**FIG 5:**
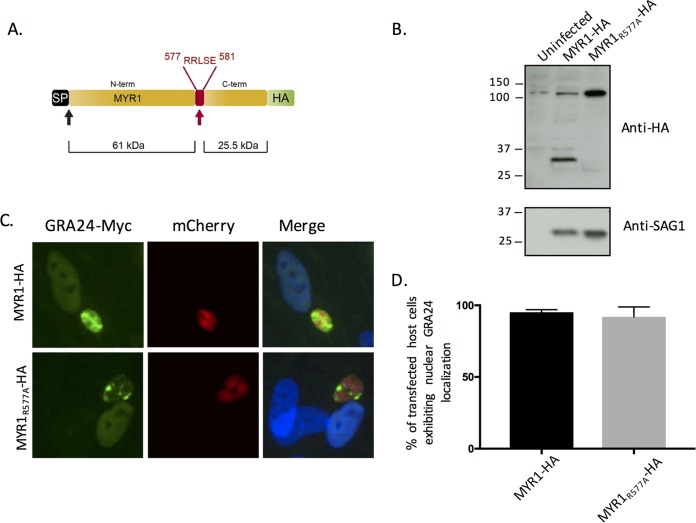
ASP5 cleavage of MYR1 at the TEXEL is not essential for MYR1 functionality. (A) The ASP5 cleavage motif in endogenously tagged RH MYR-HA was mutated through an R_577_→A change to yield the MYR1R_577A_-HA strain. (B) Lysates of uninfected HFFs or HFFs infected with the MYR1-HA or MYR1R_577A_-HA strains were probed with the indicated antibodies. (Note the presence of a host protein just above 110 kDa that cross-reacts with the anti-HA antibody.) (C) Representative images showing GRA24-Myc translocation from the parasitophorous vacuole in HFFs infected with mCherry-expressing MYR1-HA or MYR1R_577A_-HA strains. (D) Quantification of GRA24-Myc translocation. Shown are the percentages of cells with nuclear localization of GRA24-Myc after infection with MYR1-HA or MYR1R_577A_-HA tachyzoites transfected transiently with GRA24-Myc. Results are from three technical repeats for a representative of three biological repeats. The average and standard deviation (SD) are shown.

The fact that far more host genes are dependent on the presence of MYR1 versus ASP5, rather than vice versa, also strongly argues that ASP5’s cleavage of MYR1 is not essential for its role in export of effectors across the PVM. To confirm this, we mutated the ASP5 cleavage site, RRL, to ARL in the endogenously hemagglutinin (HA)-tagged MYR1-HA strain (22), generating the MYR1R_577A_-HA allele (Fig. 5A). Western blot analysis of the resulting parasites confirmed that, as previously reported (23), this change eliminates cleavage of MYR1 (Fig. 5B) as the C-terminally tagged band shifted from ~30 kDa in parasites carrying a wild-type allele of MYR1 to the uncleaved size, ~110 kDa, in the MYR1R_577A_-HA-expressing parasites. Having shown that the mutated MYR1 is not processed in the usual way, we next asked if these parasites would nonetheless show translocation of MYR1-dependent effectors into the host HFF nucleus by assessing GRA24-Myc translocation (Fig. 5C). A quantification of the results (Fig. 5D) shows clear translocation, with no significant difference compared to RH-WT-infected cells, indicating that the function of MYR1 is indeed not dependent in a detectable way upon ASP5 cleavage at the MYR1 TEXEL motif. This finding offers a partial explanation for why MYR1-dependent responses in the host cell are more numerous than ASP5-dependent responses and suggests the existence of undiscovered effector proteins that are dependent on MYR1 but not ASP5 for their export.

## DISCUSSION

The results presented here show that a majority of the transcriptomic response in HFFs infected with *Toxoplasma* tachyzoites is dependent on the presence of a functional MYR1, even more so than for ASP5, an enzyme previously shown to be necessary for the translocation of three GRA effectors, GRA16, GRA24, and TgIST (23, 25). The impact on the host for each of these three effectors has been examined individually by microarray or RNA-Seq (25, 33, 34), and thus we can compare those published transcriptomic changes with the results from our study, although differences in experimental design, including the time point examined, MOI, cell type, and method of analysis, heavily qualify such comparisons. For GRA16, Bougdour et al. demonstrated by KEGG analysis a predominant impact of GRA16’s loss on the host cell cycle and p53 pathways (33). Our data are consistent with this in that the cell cycle-related genes feature prominently in our MYR1-dependent, upregulated data set, and the p53-related GSEA gene set features prominently in our MYR1-dependent, up- and downregulated data set. Bougdour et al. also showed that the effect of *Toxoplasma* infection on host DNA repair and mismatch repair is GRA16 dependent, and we demonstrate among MYR1-suppressed genes a MYR1-dependent impact on the GSEA gene set DNA repair. Interestingly, downregulation of the p53 GSEA gene set demonstrates MYR1 dependency but not ASP5 dependency. As noted above, this discrepancy could be due to differences in experimental design; alternatively, it could indicate that there is another, MYR1-dependent, ASP5-independent effector protein that intersects in a suppressive way with the activity of p53.

While we observed the host transcriptomic response to Δ*myr1* mutants to include the published phenotype of the Δ*gra16* mutants, we did not see similar concordance with regard to the Δ*gra24* mutant. Instead, it has been reported that GRA24 alters host chemokine, cytokine, and intracellular adhesion genes, and we did not see any gene set enrichment for these clusters in the MYR1-dependent data. This result was regardless of whether the RHΔ*myr1* infection was compared to RH-WT-infected cells in the head-to-head comparison or each of the infected cultures was compared to mock-infected cells. A lack of dependence on MYR1 could be explained if tachyzoites simultaneously secrete GRA24 and one or more MYR1-dependent effector proteins that counteract the effect of GRA24. While we have presented evidence here for the existence of counteracting MYR1-dependent and MYR1-independent activities, some genes might be under the influence of two or more MYR1-dependent effectors acting with opposing effects. For instance, Braun et al. show that GRA24 upregulates CXCL10, and yet we do not find the CXCL10 gene to be upregulated in a MYR1-dependent manner (34); instead, we see more upregulation in the cells infected with RHΔ*myr1* relative to mock infection than in the RH-WT versus mock infection comparison. CXCL10 is controlled through STAT1 signaling (38), and so while GRA24 may be upregulating the CXCL10 gene, a MYR1-dependent suppressor protein may simultaneously be suppressing its expression. TgIST is a good candidate for such a suppressor as it has a single TEXEL motif and requires ASP5 to be exported from the parasitophorous vacuole. Furthermore, in the study by Franco et al. (22), we demonstrate that the suppression of type II interferon responses is MYR1 dependent, making it likely that the export of TgIST is dependent on this protein. Consistent with this, we see a strong enrichment of type I interferon responses among the GSEA gene sets suppressed in a MYR1-dependent manner as well as enrichment in interferon-related genes that are upregulated only in the cells infected with MYR1-deficient parasites but not the wild type. Again, as in the case of GRA16, we do not observe comparable GSEA results in the ASP5-dependent gene set as we do in the MYR1-dependent gene set with regard to interferon suppression: interferon suppression was only enriched in the MYR1-dependent genes. This is surprising because TgIST export is ASP5-dependent, but the fact that our data were collected on cells infected for only 6 h compared to substantially later in other studies may explain much or all of the paradox: i.e., ASP5 might be required for the export of certain effector proteins only at time points later than 6 h. Consistent with this, Gay et al. show that at 24 hpi, GSEA on cells infected with TgIST mutants shows enrichment for TNF-α responses, inflammatory responses, IL-6 via STAT3, type II interferon responses, KRAS signaling, complementation, and apoptosis (25). We find all these gene sets to be significantly enriched in the MYR1-dependent data set but not in the ASP5-dependent gene sets.

It is apparent in examining the transcriptomic analyses side by side for cultures infected with these individual effector knockouts and the MYR1 knockout how complex the control of the host cell is. IRF1 is a good example of an effect that is mediated by multiple effectors—both MYR1-dependent and MYR1-independent—based on what has been published to date about its response to *Toxoplasma*; the rhoptry kinase ROP16, a MYR1-independent effector, activates STAT1, which is a known inducer of IRF1 transcription (5). However, the activity of this activated IRF1 is blocked in a MYR1-dependent manner (22), and one effector documented to do this is TgIST (25). Thus, TgIST should counteract the STAT1 activation by ROP16 and prevent transcription of STAT1-dependent genes, giving rise to a wild-type infection phenotype for these genes that mimics the level seen with mock infection; however, when TgIST cannot be exported because of the defect in MYR1, the activation of IRF1 by ROP16 then becomes observable.

Another example of a complex interplay may be EGR1, which has been reported to be directly upregulated by GRA24. When GRA24 is knocked out, expression of EGR1 has been reported to decrease (34). In our data set, however, when MYR1 or ASP5 is deleted, EGR1 transcripts actually increase relative to infection with RH-WT, indicating the existence of a more complex level of control of the EGR1 gene via additional effectors. Specifically, the data fit a model where the MYR1-dependent activator GRA24 and suppressor TgIST operate in the context of a MYR1-independent activator, with all three targeting the same host pathways and engaging in a tug-of-war on these genes’ expression levels.

Within our data set, there are host genes whose regulation demonstrates dependence on ASP5 but not on MYR1. Because this would hint at ASP5-processed effectors that are secreted in a non-MYR1-dependent manner, we examined the expression of these genes in greater depth. There are 126 genes demonstrating an upregulation and 27 genes demonstrating a downregulation dependent upon ASP5 and not MYR1; however, although most of these genes appear to show a lower or higher expression, respectively, in cells infected with RH*Δmyr1* compared to cells infected with RH-WT, they did not meet the threshold of both 1.5-fold change and statistical significance. Hence most—perhaps all—of the host gene responses that are ASP5-dependent may, to a substantial extent, also be MYR1-dependent.

Overall, the results presented here reveal a complex interplay of *Toxoplasma* activators and suppressors, some of which are dependent on the action of ASP5 for processing and MYR1 for translocation into the infected host cell, while others appear independent of one or both of these proteins. Our data also point to a large number of host genes whose expression appears not to change upon infection but are actually kept at uninfected levels through the use of counteracting activities; some of these activities may be pathogen-associated molecular patterns or other molecules that stimulate a particular response that are simultaneously countered by MYR1-dependent effectors working to maintain an environment conducive to parasite growth. The data sets presented here provide new insight into the role of existing effector proteins and posit the existence of yet undiscovered activities. In clarifying the existence and role of such molecules, we can better understand how *Toxoplasma* tachyzoites control infected host cells to their advantage.

## MATERIALS AND METHODS

### Parasite strains and cell culture.

Toxoplasma gondii strains were maintained by growth in confluent primary human foreskin fibroblasts (HFFs) in Dulbecco’s modified Eagle’s medium (DMEM; Invitrogen, Carlsbad, CA) with 10% fetal bovine serum (FBS; HyClone, Logan, UT), 2 mM glutamine, 100 U/ml penicillin, and 100 μg/ml streptomycin (cDMEM) at 37°C in 5% CO_2_.

The following type I strains were used: RH, RHΔ*myr1*, and RHΔ*myr1*::*MYR1* (22), RHΔ*asp5* and RHΔ*asp5*::*ASP5* (23), RH MYR1-HA (RHΔ*ku80*) (22), and RH MYR1R_577_A-HA (RHΔ*ku80*). The RH MYR1R_577A_-HA (RHΔ*ku80*) strain was generated by amplifying a gBlock (IDT) in which the codon for arginine at residue 577 of MYR1 (CGG) is mutated to one for alanine (GCT). The resulting product was purified and cotransfected into RH MYR1-HA with pSAG1:U6-Cas9:sgMYR1 2081, which was generated by site-directed mutagenesis (NEB) on the pSAG1:U6-Cas9:sgUPRT plasmid (39). Parasites transiently expressing Cas9-GFP (green fluorescent protein) were enriched by fluorescence-activated cell sorter (FACS) sorting at 24 h postinfection, and the resulting population was allowed to infect HFFs in DMEM. The parasites were cloned by limiting dilution and screened for endogenous mutation at the correct locus using PCR and sequencing.

### Parasite transfection and immunofluorescence imaging.

All transfections were performed using the BTX EMC600 electroporation system (Harvard Apparatus, Inc.) or Amaxa 4-D Nucleofector (Lonza) model. Tachyzoites were mechanically released in phosphate-buffered saline (PBS), pelleted, and resuspended in solution for transfection. After transfection, parasites were allowed to infect HFFs in DMEM. Transfections with the BTX EMC600 model were performed using 5 × 10^6^ to 10 × 10^6^ parasites and 15 to 25 µg DNA in Cytomix (10 mM KPO_4_ [pH 7.6], 120 mM KCl, 5 mM MgCl_2_, 25 mM HEPES, 2 mM EDTA, 150 µM CaCl_2_). Transfections with the Amaxa 4-D model were performed using 2 × 10^6^ to 4 × 10^6^ parasites in 20 µl P3 solution with 2 to 5 µg DNA. Effector translocation assays were performed by transiently transfecting pHTU-GRA24-3×Myc (23), into tachyzoites, infecting HFF monolayers in DMEM, and fixing monolayers with 4% formaldehyde for 15 min (at room temperature) at 16 to 24 hpi. Fixed samples were rinsed once with PBS, permeabilized with 0.2% Triton X-100 (TTX-100) for 20 min, blocked for 1 h at room temperature, and then incubated with rabbit anti-Myc tag antibody (CST) and goat polyclonal Alexa Fluor 488-conjugated anti-rabbit secondary antibody. Vectashield with DAPI (4′,6-diamidino-2-phenylindole) stain (Vector Laboratories) was used to mount the coverslips on slides, and fluorescence was detected using an epifluorescence microscope.

### RNA extraction, library preparation, and sequencing.

HFFs were serum starved for 24 h before infection by growth in DMEM containing 0.5% serum. They were then infected with the indicated line of tachyzoites at an MOI of 5, and at 6 hpi, 1 ml TRIzol reagent (Invitrogen) was added to each T25 flask, and the cells were scraped. Lysates were collected into RNase/DNase-free Eppendorf tubes and frozen at −20°C. Total RNA was extracted following the manufacturer’s instructions, with some modifications. Briefly, frozen samples were thawed on ice, and 0.2 ml chloroform was added to TRIzol suspensions, which were then mixed by inversion 10 times, and incubated for 5 min. Tubes were then spun at 12,000 rpm for 15 min at 4°C. RNA in the aqueous phase was transferred into a fresh tube, and 0.5 ml absolute isopropyl alcohol was added. Each tube was inverted three times and incubated at 4°C for 10 min. They were then spun at 12,000 rpm for 20 min at 4°C. After decanting of the supernatants, RNA pellets were washed with 1 ml 75% ethanol. Tubes were mixed by inverting 10 times and then spun at 12,000 rpm for 20 min at 4°C. Supernatants were removed and the RNA pellets were air dried in open tubes for approximately 10 min. The RNA pellets were resuspended in 30 μl RNase-free diethyl pyrocarbonate (DEPC)-water. RNA samples were submitted to the Stanford University Functional Genomic Facility (SFGF) for purity analysis using the Agilent 2100 Bioanalyzer. Multiplex sequencing libraries were generated with an RNA Sample Prep kit (Illumina) according to the manufacturer’s instructions and pooled for a single high-throughput sequencing run using the Illumina NextSeq platform (Illumina Nextseq 500 model instrument).

### RNA-Seq read mapping and differential expression analysis.

Raw reads were uploaded onto the CLC Genomics workbench 8.0 (Qiagen) platform for independent alignments against the human genomes (Ensembl.org/ hg19) and *Toxoplasma* type I GT1 strain (ToxoDB-24, GT1 genome). All parameters were left at their default values. The number of reads mapping to exons in the human and *Toxoplasma* genomes and the percentage of reads mapping to these genomes are listed in Table 1.

The number of total reads mapped to each genome was used to determine the RPKM (reads per kilobase of transcript per million mapped reads). The SAMseq (40) package for the R platform was used to identify genes with significant changes between two samples. To identify genes with statistically different expressions between samples, we set the delta (Δ) value at a 10% false-discovery rate (FDR) with a *q* value of <0.05. Among these genes, only those with average RPKM ratios of ≥1.5 were counted as changed in expression.

Heat maps were generated using Gene E (https://software.broadinstitute.org/GENE-E/index.html). Venn diagrams were created using BioVenn (41).

### GSEA.

GSEA software, available through the Broad Institute at http://www.broadinstitute.org/gsea/index.jsp, was the enrichment analysis software we used to determine whether defined sets of differentially expressed human genes in our experiment show statistically significant overlap of gene sets in the curated Molecular Signatures Databases (MsigDB) Hallmark gene set collection. We used the cutoff of an FDR *q* value of <10^−2^. The list of genes that are found in the gene sets presented is displayed in Tables S4 to S8.

10.1128/mBio.02401-17.5 TABLE S4  Genes expressed higher when infected with RH (A) or Δ*myr1* parasites (B) versus mock infected. The table provides the list of genes accounting for the gene sets shown in Figure 2. Download TABLE S4, XLSX file, 0.1 MB. Copyright © 2018 Naor et al. 2018 Naor et al. This content is distributed under the terms of the Creative Commons Attribution 4.0 International license.

10.1128/mBio.02401-17.6 TABLE S5  Genes expressed lower when infected with RH (A) or Δ*myr1* parasites (B) versus mock infected. The table provides the list of genes accounting for the gene sets shown in Figure 2. Download TABLE S5, XLSX file, 0.1 MB. Copyright © 2018 Naor et al. 2018 Naor et al. This content is distributed under the terms of the Creative Commons Attribution 4.0 International license.

10.1128/mBio.02401-17.7 TABLE S6  Genes expressed higher when infected with RH versus Δ*myr1* (A) or Δ*asp5* (B) parasites. The table provides the list of genes accounting for the gene sets shown in Figure 3. Download TABLE S6, XLSX file, 0.1 MB. Copyright © 2018 Naor et al. 2018 Naor et al. This content is distributed under the terms of the Creative Commons Attribution 4.0 International license.

10.1128/mBio.02401-17.8 TABLE S7  Genes expressed lower when infected with RH versus Δ*myr1* (A) or Δ*asp5* (B) parasites. The table provides the list of genes accounting for the gene sets shown in Figure 3. Download TABLE S7, XLSX file, 0.1 MB. Copyright © 2018 Naor et al. 2018 Naor et al. This content is distributed under the terms of the Creative Commons Attribution 4.0 International license.

10.1128/mBio.02401-17.9 TABLE S8  Genes expressed lower when infected with Δ*myr1* versus RH parasites but not differentially expressed when comparing infection with RH parasites and mock infected. The table provides the list of genes accounting for the gene sets shown in Figure 4. Download TABLE S8, XLSX file, 0.1 MB. Copyright © 2018 Naor et al. 2018 Naor et al. This content is distributed under the terms of the Creative Commons Attribution 4.0 International license.

### Accession number(s).

The RNA-Seq data files have been deposited in GEO under accession no. GSE109830.
