# Supplementary figures and images for: MYR1-Dependent Effectors Are the Major Drivers of a Host Cell’s Early Response to Toxoplasma, Including Counteracting MYR1-Independent Effects
Source: mBio. 2018 Apr 3;9(2):e02401-17. doi: 10.1128/mBio.02401-17 (PMC5885026; doi:10.1128/mBio.02401-17)

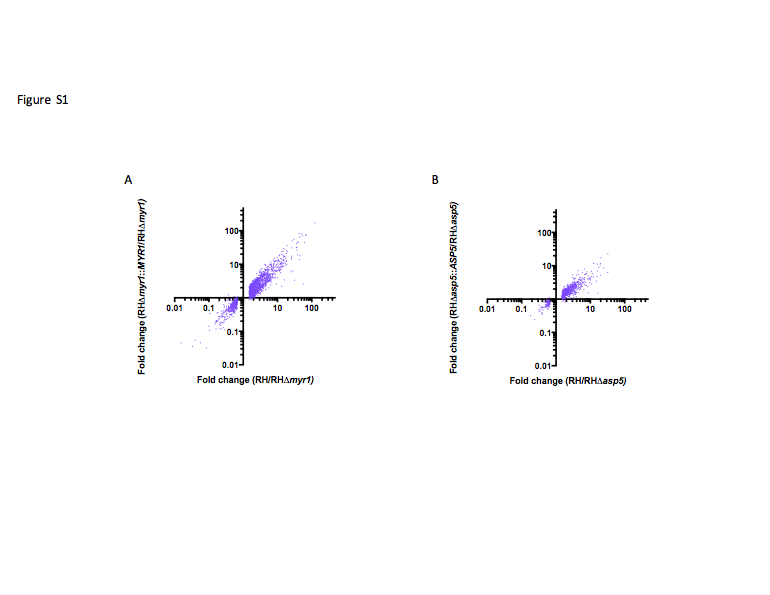

Supplement: FIG S1 [file mbo002183813sf1.tif]
